# Comparative Transcriptome Analysis Using High Papaverine Mutant of *Papaver somniferum* Reveals Pathway and Uncharacterized Steps of Papaverine Biosynthesis

**DOI:** 10.1371/journal.pone.0065622

**Published:** 2013-05-30

**Authors:** Sumya Pathak, Deepika Lakhwani, Parul Gupta, Brij Kishore Mishra, Sudhir Shukla, Mehar Hasan Asif, Prabodh Kumar Trivedi

**Affiliations:** CSIR-National Botanical Research Institute (CSIR-NBRI), Rana Pratap Marg, Lucknow, India; Cankiri Karatekin University, Turkey

## Abstract

The benzylisoquinoline alkaloid papaverine, synthesized in low amount in most of the opium poppy varieties of *Papaver somniferum*, is used as a vasodilator muscle relaxant and antispasmodic. Papaverine biosynthesis remains controversial as two different routes utilizing either (S)-coclaurine or (S)-reticuline have been proposed with uncharacterized intermediate steps. In an attempt to elucidate papaverine biosynthesis and identify putative genes involved in uncharacterized steps, we carried out comparative transcriptome analysis of high papaverine mutant (*pap1*) and normal cultivar (BR086) of *P. somniferum*. This natural mutant synthesizes more than 12-fold papaverine in comparison to BR086. We established more than 238 Mb transcriptome data separately for *pap1* and BR086. Assembly of reads generated 127,342 and 106,128 unigenes in *pap1* and BR086, respectively. Digital gene expression analysis of transcriptomes revealed 3,336 differentially expressing unigenes. Enhanced expression of (S)-norcoclaurine-6-O-methyltransferase (6OMT), (S)-3′-hydroxy-N-methylcoclaurine 4′-O-methyltransferase (4′OMT), norreticuline 7-O-methyltransferase (N7OMT) and down-regulation of reticuline 7-O-methyltransferase (7OMT) in *pap1* in comparison to BR086 suggest (S)-coclaurine as the route for papaverine biosynthesis. We also identified several methyltransferases and dehydrogenases with enhanced expression in *pap1* in comparison to BR086. Our analysis using natural mutant, *pap1*, concludes that (S)-coclaurine is the branch-point intermediate and preferred route for papaverine biosynthesis. Differentially expressing methyltransferases and dehydrogenases identified in this study will help in elucidating complete biosynthetic pathway of papaverine. The information generated will be helpful in developing strategies for enhanced biosynthesis of papaverine through biotechnological approaches.

## Introduction

Benzylisoquinoline alkaloids (BIAs) form one of the largest and most diverse groups of alkaloids with about 2500 members. Opium poppy (*Papaver somniferum*) considered as the only source for several high-value pharmaceutically important BIAs including the narcotic analgesic morphine, antitussive codeine, the potential anti-cancer drug noscapine, the antimicrobial agent sanguinarine and the vasodilator papaverine. The papaverine, synthesized in low amounts in most of the opium poppy varieties, is also used as smooth muscle relaxant [1], erectile dysfunction [2], treatment of intestinal and urinary tract spasms [3], migraine headaches [4] and schizophrenia [5]. All these effects are attributed to its inhibitory effect on phosphodiesterases by increasing cAMP levels [5], [6].

BIA biosynthesis begins with the condensation of two L-tyrosine derivatives, 4-hydroxyphenylacetaldehyde (4-HPAA) and dopamine, catalysed by norcoclaurine synthase (NCS) which yields (S)-norcoclaurine [7], [8]. Sequential methylations and hydroxylation with array of enzymes produce (S)-reticuline [9]–[11]. (S)-reticuline acts as the central intermediate which is diverted to different branches for the biosynthesis of different types of BIAs. Out of several branches, biosynthesis pathway and enzymes leading to morphinans is most characterized though many intermediate steps and regulatory mechanisms are still uncharacterized [12]–[17]. Recently, established morphine biosynthesis pathway has been reconstructed using high throughput analysis involving transcriptome sequencing and gene silencing approaches [18].

In case of papaverine biosynthesis pathway, very limited and controversial information is available. Feeding experiments suggested that (S)-reticuline and nororientaline as well as norreticuline to be possible pathway intermediates [19], [20]. A few putative pathways have been proposed with these molecules as intermediates. The first proposed pathway, NH pathway (involving N-desmethyl intermediates), for papaverine biosynthesis utilizes (S)-coclaurine with the involvement of 3′-hydroxylase, and norreticuline 7-O-methyltransferase (N7OMT) [21]. The second proposed pathway, NCH_3_ (involving several N-methylated intermediates), pathway begins with the conversion of (S)-reticuline to (S)-laudanine catalyzed by reticuline 7-O-methyltransferase (7OMT) [22]. As a result of subsequent 3′-O-methylation, N-demethylation and dehydrogenation papaverine is synthesized [23]. These subsequent 3′-O-methylation, N-demethylation and dehydrogenation steps are essential in both routes to yield papaverine. Recently, through transcriptome sequencing and virus-induced gene silencing (VIGS) it has been suggested that intermediate steps from both previously reported pathways operate for papaverine biosynthesis [24]. Contrary to this, the same group reported that the major pathway to papaverine biosynthesis involves N-desmethyl intermediates and does not primarily proceed via (S)-reticuline [25]. Taken together, these studies suggest that papaverine biosynthesis still remains controversial more than a century after the pathway was first suggested.

The major limitation in understanding papaverine biosynthesis and other BIAs is due to limited genomic information about this plant. In different plants, two strategies, one to establish complete genome and other to generate ESTs have been helpful in understanding pathways and networks leading to biosynthesis of primary and secondary metabolites as well as plants behavior to different stresses. At present, total genome sequencing of poppy plant has not been initiated due to large genome size (7.4 Gbp) [26]. Initially, EST datasets have been utilized for gene discovery and to elucidate alkaloid biosynthesis pathway to some extent [11], [17]. Use of Next Generation Sequencing (NGS) technologies led to the establishment of transcriptomes of few poppy cultivars with differential BIA content and discovery of a number of previously unidentified genes [25], [27]. Recently, analysis of mutants for elucidation of biosynthesis pathways for specific alkaloids had been emphasized in addition to stepwise verification [18], [28]. Using this approach, metabolic block in *top1* (thebaine oripavine poppy 1) mutant, which is defective in 6-O-demethylation of thebaine and oripavine [29], has helped in identifying genes involved in thebaine biosynthesis [17]. Studies also suggest that mutants selectivity allow exploration of targeted redundant pathway branches and may enable enhanced understanding for efficient production of high value compound using biotechnological approaches [18].

Here, we have characterized *pap1* (naturally selected high papaverine mutant 1) mutant of opium poppy, which accumulates papaverine up to 6% of the latex with visible phenotype of white latex in comparison to normal cultivar (BR086) with 0–0.5% papaverine of normal pink latex. We established complete transcriptome of *pap1* and BR086 using high-throughput 454 Genome Sequencer (GS) FLX platform with an objective to identify genes involved in the biosynthesis of papaverine. We report a number of differentially expressing unidentified transcripts between *pap1* and BR086. Using this data we have identified members of methyltransferase (MT) and dehydrogenase gene families. Expression of differentially expressed genes has been validated through Real-Time PCR. On the basis of identified genes, we propose that identified genes may be potential candidates for elucidating papaverine biosynthesis in *P. somniferum*.

## Materials and Methods

### Plant material, cDNA library construction and sequencing

Peduncle tissue (stem, 2 cm below the capsule) of prehook stage of *pap1* mutant and BR086 cultivar of *Papaver somniferum* L. were collected from four plants grown in the experimental plot of the Institute. Frozen tissues were ground to a fine powder in liquid nitrogen and total RNA was extracted using Spectrum Plant Total RNA Kit (Sigma–Aldrich, USA) and treated with RNase free DNaseI (Ambion, USA) according to manufacturer’s instructions. The quality and quantity of total RNA were analyzed by agarose gel and spectrophotometric analysis (ND-1000 Nanodrop, NanoDrop Technologies, USA). The equal amount of total RNA from four different preparations was pooled and used for further processing for transcriptome sequencing. Double-stranded (ds) cDNA library was prepared using pooled total RNA and double stranded cDNA synthesis kit (Invitrogen, Carlsbad, CA) as per manufactures recommendations. Quantity as well as quality of (ds) cDNA library was checked on Agilent 2100 Bioanalyzer DNA chip (Agilent Technologies Inc., Santa Clara, CA). Random fragments of about 250–800 bp in length of (ds) cDNA were generated by nebulization and purified using QIAquick PCR purification spin columns (Qiagen, USA). Adapter ligated (ds) cDNA libraries were prepared using fragments above 300 bp according to manufacturer’s instruction (Roche, USA). (ds) cDNA fragments were denatured to generate single-stranded cDNA fragments, which were amplified by emulsion PCR for further sequencing according to manufacturer’s instructions (Roche, USA). The sequencing of cDNA libraries was performed on a 454-GS FLX sequencing platform (454 Life Sciences, Roche, USA) using GS FLX Titanium Kit.

### De novo assembly and sequence annotation

Reads from *pap1* and BR086 libraries were processed and trimmed for weak signals by GS FLX pyrosequencing software to yield high-quality (HQ) (≥99.5% accuracy of single-base reads) sequences. The HQ reads were assembled using Roche GS Assembler (version 2.5.3) with 40 base pair overlap and 95% identity for independent libraries forming contigs and singletons. The contigs and singletons of both libraries (*pap1* and BR086) were tagged and assembled together for the purpose of quantifying differential expression of unigenes. Total unigenes formed in different libraries were pooled and annotated using standalone version of BLASTx program against protein Nr database (http://www.ncbi.nlm.nih.gov; released on 06/23/2009), Arabidopsis protein database at The Arabidopsis Information Resource (TAIR; http://www.arabidopsis.org; version Tair9) and NCBI-CDD (Conserve Domain Database) database with an E-value cut-off of 10^−5^ and extracting only the top hit for each sequence.

### Functional characterization and biological pathways assignment

To assign function to each unigene, gene ontology (GO) analysis was performed using GO annotation in online TAIR Database (http://www.arabidopsis.org/), which classified unigenes under the categories of Cellular component, Molecular Function and Biological Process. The TAIR IDs of all the unigenes (contigs and singletons) from *pap1* and BR086 were retrieved from TAIR9 annotation. Each annotated sequence may have more than one GO term, either assigned in the different GO categories or in the same category [30] **.** To gain an overview of gene pathway networks, the assignment of unigenes from combined transcriptome into metabolic pathways were mapped according to the Kyoto Encyclopedia of Genes and Genomes (KEGG) [31]. KO numbers were assigned to unique sequences, based on the BLASTx search of protein databases, using a cutoff E value 10^−5^. The output of KEGG analysis includes KEGG orthology (KO) assignments and KEGG pathways (http://www.genome.jp/kegg/) that are populated with the KO assignments.

### Digital gene expression profiling

Relative gene expression level for different unigenes was calculated using number of reads per unigenes in combined assembly of *pap1* and BR086 transcriptome. To carry out this, the contigs and singletons of *pap1* and BR086 libraries were tagged, assembled together and annotated. Transcripts per million (tpm) were calculated for each contig formed for further analysis. Relative expression values of unigenes in *pap1* and BR086 samples from combined assembly were computed by dividing its 454-sequence reads by the total 454-sequence reads in both the samples. Differential expression of genes was calculated by using R value and fold change. To calculate the threshold R value, 1000 datasets for each library was generated according to the random poisson distribution as described in [32]. Log 2 converted values for each unigene was subjected in MeV (Multiple Experiment Viewer, Java tool for genomic data analysis, version 4.8.1). R value ≥8 and fold change ≥2 for a unigene was considered as differential expression. Hierarchical clustering (HCl) of log-transformed expression data was carried out using Euclidean Distance Matrix and Average linkage clustering method [33].

### Real-time PCR analysis

Expression of putative genes involved in papaverine biosynthesis was analyzed through qRT-PCR using Real-Time PCR Detection System (ABI 7500, Applied Biosystems, USA) and fast SYBR Green PCR Master Mix (Applied Biosystems, Warrington, UK) to validate 454 sequencing data. Each PCR reaction was set up in total 20 µl volume containing 10 µl of Fast SYBR green master mix, 10 ng of cDNA sample prepared using RevertAid H minus first strand cDNA synthesis Kit (Fermantas, Life Sciences, Ontario, Canada) and gene-specific primers (Table S1). The PCR cycling conditions were as follows: 50°C for 2 min, 95°C for 2 min for initial denaturation followed by 40 cycles of 95°C for 15 s and 60°C for 60 s. Each time, reaction sets also incorporated a no-template control. We employed probes specific for the actin as references. The qPCR data was analysed with the ΔΔCT method using reference gene. For each sample, the mRNA levels of target genes were normalized to that of the actin mRNA. All the experiments were repeated using three biological replicates and the data were analyzed statistically (± Standard Deviation).

### Alkaloid content estimation

Opium latex from capsules from field matured plants of *pap1* and BR086 were collected and stored in DMSO for alkaloid extraction. Solvent containing extracted alkaloids was recovered and estimation of various alkaloids was carried out through separation using High Pressure Liquid Chromatography following the standard method [34], [35]. The average content of each alkaloid of at least three samples was calculated and data are expressed as mean +/−standard deviation (S.D.).

## Results and Discussion

### Enhanced papaverine biosynthesis in *pap1* mutant

**Figure 1:**
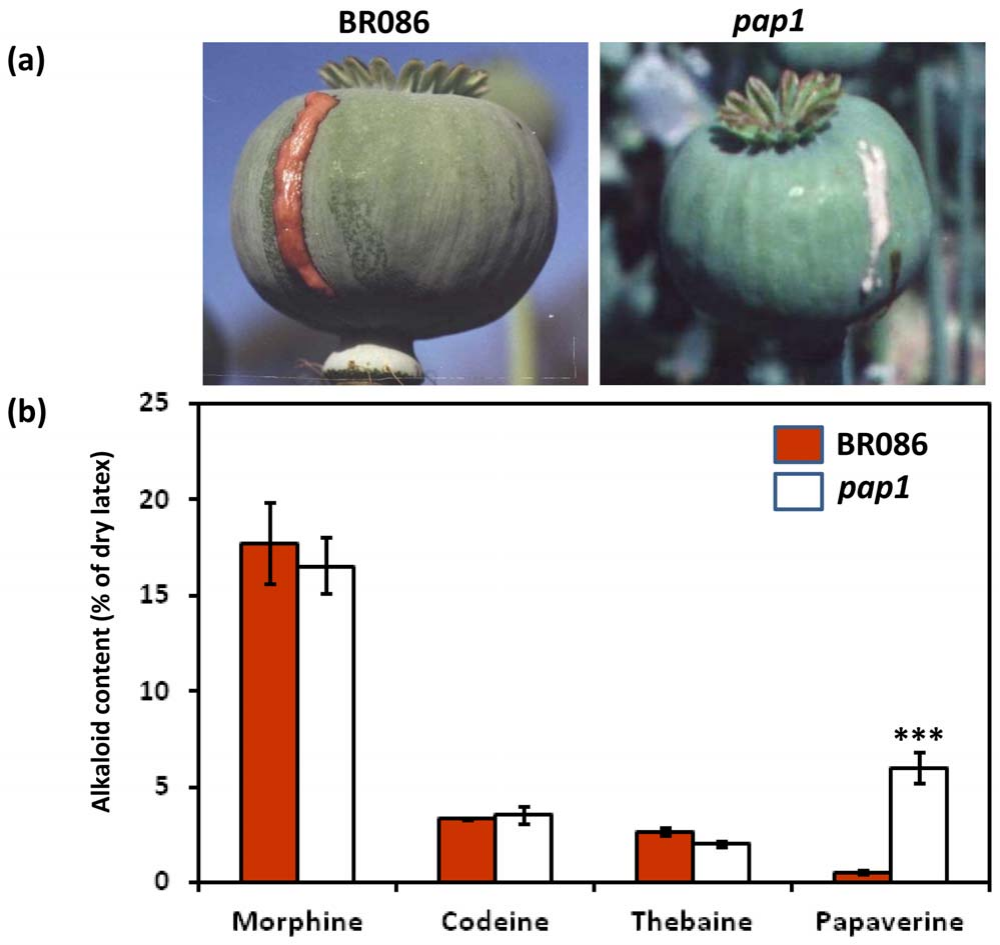
Phenotype of fresh latex and specific alkaloid content in ***pap1*** **and BR086.** Fresh latex of BR086 is brown or red whereas *pap1* exuded white latex (a). Content of specific alkaloids in latex was measured in *pap1* and BR086 (b) using High Pressure Liquid Chromatography. *** indicate values that differ significantly from BR086.

Distinct phenotypes with respect to colour of fresh latex was observed upon lancing capsules of *pap1* mutant in comparison to BR086 of *P.somnifera.* In contrast to pink latex produced in BR086, *pap1* exuded white latex (Figure 1a). Earlier, Khanna and Shukla (1991) [36] studied inheritance of papaverine content in selected opium poppy lines and established positive correlation between white fresh latex as morphological marker and high papaverine content. Fresh white latex exudation and papaverine content has also been suggested to be correlated with gene action through crossing of high papaverine line with BR086 line and analysis of six subsequent generations [37]. As naturally selected *pap1* mutant exudes white latex on lancing, analysis of major morphinans (thebaine, codeine and morphine) and papaverine was carried out in *pap1* mutant and BR086 (Figure 1b). No significant difference in thebaine, codeine and morphine content in latex was observed between *pap1* and BR086. However, papaverine content was more than 12-fold higher (6% of the dried latex) in *pap1* in comparison to BR086 line (0.48% of the latex). As *pap1* mutant accumulated significantly high papaverine content without changes in accumulation in morphinans, we hypothesized that this may serve as good model to explore and elucidate papaverine biosynthesis pathway.

It has been suggested that early stages of BIA biosynthesis *in P. somniferum* occur in parenchyma cells associated with the vascular bundle and surrounding laticifers in aerial plant parts. After synthesis alkaloids accumulate into laticifers of capsule [38]. In addition to capsule, stem tissue that lies within 2–4 cm of the capsule is also a rich source of latex containing alkaloids. Immunolocalization of the alkaloid biosynthetic enzymes and major latex protein (MLP) from this area of stem suggested that complete machinery involved in the biosynthesis of alkaloids resides in this tissue [38]. Therefore, we used stem tissue below 2 cm of capsule (peduncle) for further transcriptomic analysis. Other studies have also utilized same tissue to carryout transcriptome analysis of different poppy varieties [29], [39].

### Transcriptome sequencing and sequence assembly

**Figure 2:**
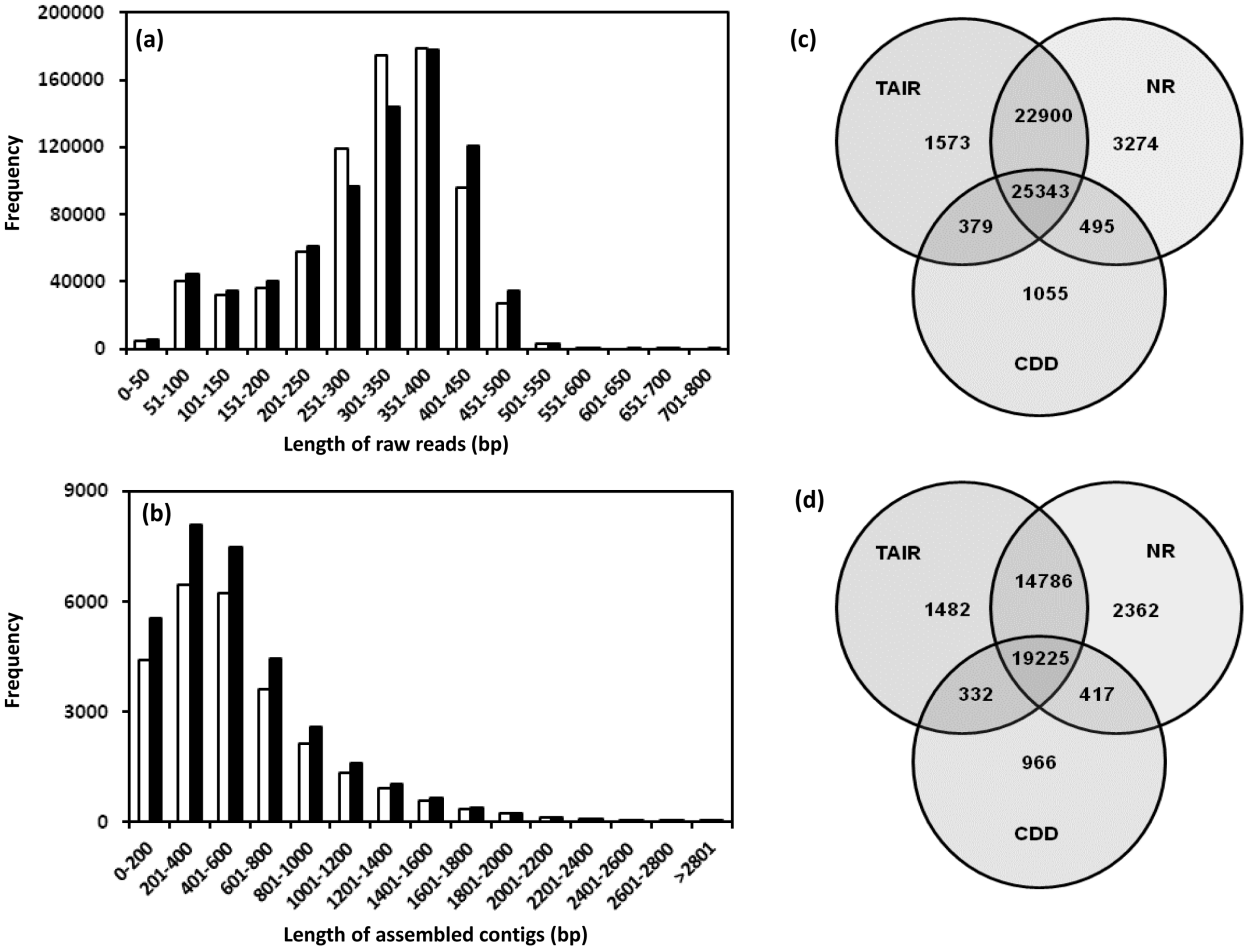
Sequencing reads, assembly and annotation. Size distribution of 454 high-quality reads (a) and assembled contigs (b). Black and white bars represents reads and contigs from *pap1* and BR086 transcriptome data respectively. Annotation of unigenes of *pap1* (c) and BR086 (d) was carried out against TAIR, NR and CDD databases. Out of all annotated unigenes, 25,343 and 19,225 unigenes from *pap1* and BR086, respectively, had common BLAST hits to annotated proteins by different databases.

cDNA libraries from mRNA from peduncle tissue of *pap1* and BR086 of *P. somniferum* were sequenced using half plate run for each on a 454-GS FLX Titanium platform. After initial quality filtering through trimming adaptor sequences and removing reads shorter than 50 bp, sequencing run yielded 770,126 (238 Mb) and 763,579 (238 Mb) high-quality (HQ) expressed sequence tags for *pap1* and BR086 line respectively (Table 1). The raw sequences files SRR403064 (*pap1*) and SRR403061 (BR086) were deposited at NCBIs Short Read Archive under SRA049668. Approximately 90% of the reads obtained were between 200 and 600 bp from both transcriptomes. De novo assembly of reads led to the construction of 26,657 and 32,313 contigs with average lengths of 579 and 557 bases from *pap1* and BR086 cultivar, respectively (Table 1). The size distribution of the 454-reads and assembled contigs is shown in Figure 2 (a and b). Among all the contigs, 46% *pap1* and 44% BR086 contigs with lengths longer than 500 bp were considered large contigs. Apart from assembly as contigs, 100,685 and 73,815 reads with average length of more than 274 bp remained as singletons. GC content of unigenes from *pap1* and BR086 was approximately 40% (Table 1).

**Table 1:** Summary of 454 sequencing and assembly for *pap1* mutant and BR086 line of *Papaver somnifera.*

Features	*pap1* mutant	BR086 line
HQ reads	770126 (238514475 bp)	763579 (238330001 bp)
Average HQ read length	309.7 bp	312.1 bp
Reads Assembled as contigs	561244	561677
Number of contigs	26657 (15443669 bp)	32313 (18007370 bp)
Average length of contigs	579.3 bp	557.3 bp
Range of contigs length	100–5416 bp	100–5774 bp
Contigs above 200bp	22245	26770
GC content	40.80%	40.80%
Number of Singletons	100685 (28378228 bp)	73815 (20245592 bp)
Average length of singletons	281.9	274.3
Range of singleton lengths	50–695 bp	50–548 bp
Singletons above 200 bp	77764	52960
GC content	39.90%	39.50%

### Functional annotation and classification of transcriptomes

The assembled sequences, contigs and singletons, from *pap1* and BR086 line were compared with the sequences in three major public protein databases (NR, TAIR and CDD) using the Basic Local Alignment Search Tool X (BLASTX) algorithm with an E-value cutoff of 10^−5^. A total of 127,342 and 106,128 unigenes (contigs and singletons; Table 1) from *pap1* and BR086 respectively were used for annotation using NR, TAIR and CDD database (Tables S2–S7). Collectively, a total of 55,019 (43.2%) and 39,570 (37.28%) unigenes from *pap1* and BR086 transcriptome data respectively were annotated using these databases (Table S8). Out of all, 25,343 and 19,225 unigenes from *pap1* and BR086, respectively, had common BLAST hits to annotated proteins in NR, TAIR and CDD (Figure 2c and 2d). More than 38.47 and 30.95 percent of singletons from *pap1* and BR086 also got annotated using these protein databases. The large number of singleton sequences with BLAST matches indicates that unassembled sequences still provide an abundance of valuable sequence information and improve overall transcriptome coverage.

**Figure 3:**
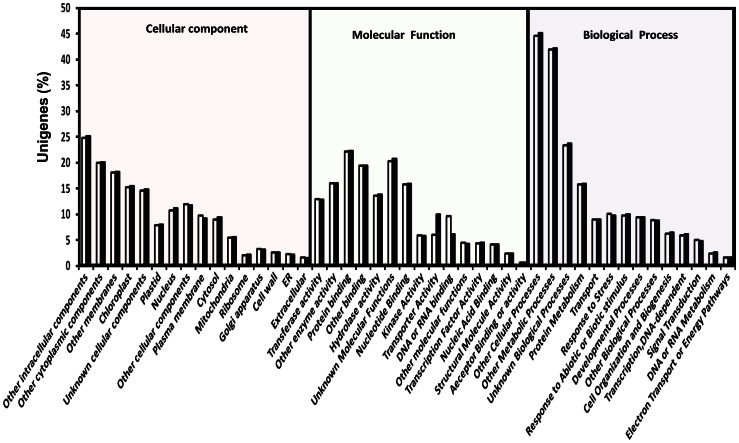
Histogram of gene ontology classification. The results are summarized in three main categories: Biological Process, Cellular Component and Molecular Function. Bars represent the assign percent of unigenes in *pap1* (white bars) and BR086 (black bars) with BLAST matches in the TAIR9 database to each GO term.

Gene Ontology (GO) assignments describe gene products in terms of their associated molecular functions, biological processes and cellular components. To assign putative functional roles to the unigenes from *pap1* and BR086, BLASTX in Arabidopsis (TAIR) proteome was carried out to provide the GO annotation. Of the assigned GO terms, 17,140 (*pap1*) and 16,615 (BR086) unigenes were to biological processes, 17,084 (*pap1*) and 16,600 (BR086) unigenes to molecular function, as well as 15,294 (*pap1*) and 14,752 (BR086) unigenes to cellular components, indicating a large functional diversity of genes in the transcriptomic data (Figure S1). Among these, 7,654 and 7,428 unigenes in *pap1* and BR086, respectively, had an assignment in all three categories. The remaining unigenes failed to obtain a GO term, largely due to their uninformative (e.g. ‘unknown’, ‘putative’, or ‘hypothetical’ protein) description. The even distribution of assignments of proteins to more specialized GO terms further indicates that the *P.somnifera* 454 sequences obtained from *pap1* and BR086 represent proteins from a diverse range of functional classes. These three main categories of the GO classification have been further divided in 45 functional groups (Figure 3). In each of the three main categories of the GO classification, 'other metabolic process', 'other cellular processes', and 'unknown biological process ' terms are dominant. In the biological process category, a large number of genes were annotated to 'other cellular and metabolic processes', 'response to abiotic or biotic stimulus', and response to stress. Hence, established transcriptome from *pap1* and BR086 should substantially aid the discovery of novel genes involved in the biosynthesis of secondary natural products including BIAs. No major difference in number of unigenes in different GO terms was observed in *pap1* and BR086 cultivar.

### Features of *pap1* and BR086 combined assembly

**Figure 4:**
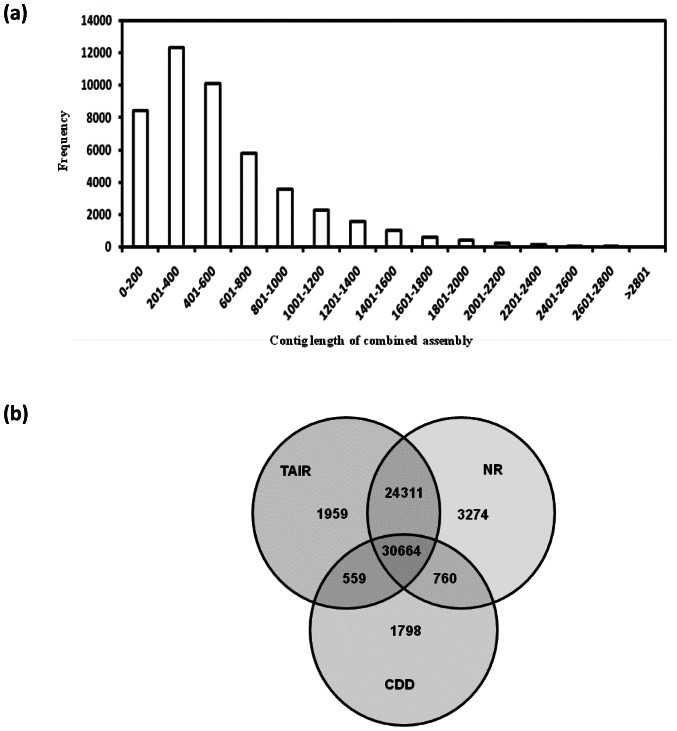
Combined assembly and annotation. Size distribution of combined assembled contig length and frequency (a) using high–quality reads from *pap1* and BR086. Annotation statistics (b) of unigenes of combined assembly queried against TAIR, NR and CDD databases.

High-quality (HQ) expressed sequence tags 770,126 and 763,579 for *pap1* and BR086 line, respectively, were tagged, pooled and assembled together to get more insight about complete poppy transcriptome. This was carried out with the aim to get larger contigs, to increase depth and to analyze differential gene expression (DGE) for specific-unigenes. HQ reads from both transcriptomes assembled into 533,705 unigenes consisting of 46,846 contigs with average length 567.5 bp and 118,841 singletons with average length of 266.2 bp. Length frequency distribution of contigs clustered between 100–4379 bp and most of the contigs ranged in length from 400 to 1200 bp (Figure 4a). The length of contigs and the number of reads assembled into that contig in combined assembly are more in comparison to independent assemblies of the *pap1* and BR086 (Table 1; Fig. 2b).

**Figure 5:**
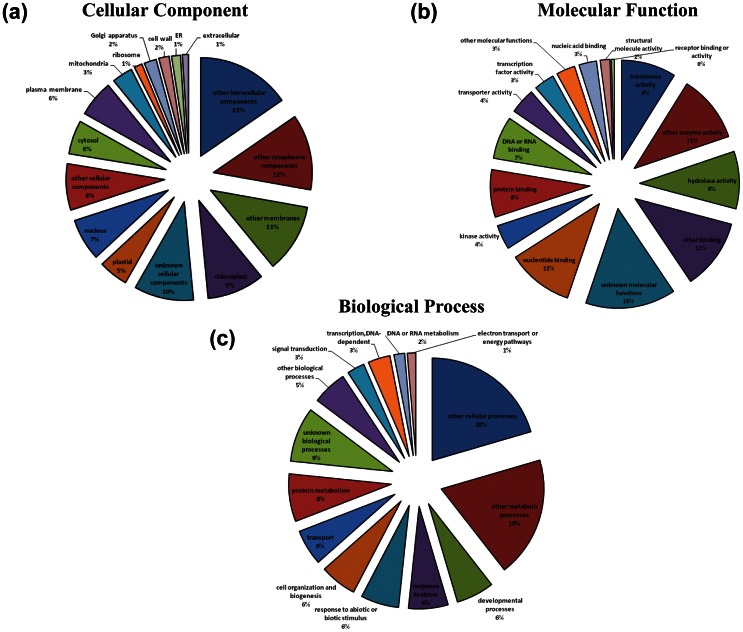
Gene ontology classification of unigenes from combined assembly of ***pap1*** and BR086. The results are summarized in three main categories: Biological Process (a), Cellular Component (b) and Molecular Function (c).

Unigenes from combined assembly of *Pap1* and BR086 were annotated with different databases (TAIR, NR, CDD) using the Basic Local Alignment Search Tool X (BLASTX) algorithm with an E-value cutoff of 10^−5^. A total 57,493 unigenes (22,213 contigs and 35,280 singletons) were annotated with TAIR database. In NR and CDD database, 59,438 and 33,781 unigenes were annotated, respectively. Common annotation for 33,664 unigenes was provided by all three databases (Figure 4b). The best hit for each unigene queried against the TAIR database from combined assembly was utilized to assign functional GO annotation in terms of biological process (17,140), molecular function (17,271) and cellular component (15,421) groups. These three main categories of the GO classification have been divided in several functional groups and subgroups (Figure 5). Three main categories of the GO classification includes molecular function (Figure 5a), cellular component (Figure 5b) and biological process (Figure 5c). In the biological process category, a large number of genes were annotated to 'other cellular and metabolic processes', 'transferase activity' and 'enzyme activity'. Several set of unigenes were associated with unknown metabolic processes and unknown cellular component. These were further categorized in several subgroups (Table S9). In these subgroups, vast majority of unigenes were found to be involved in sub cellular control of metabolic processes. A total of 7,738 unigenes were common in all three GO categories (Figure S2).

Functional classification and pathway assignment was performed by the Kyoto Encyclopedia of Genes and Genomes (KEGG). To identify the biological pathways that are functional in poppy, 57,493 annotated sequences from combined assembly were mapped to the reference canonical pathways in KEGG. In total, all contigs and singleton sequences of poppy were assigned to 124 KEGG pathways. Interestingly, 3,665 unigenes were found to be involved in biosynthesis of various secondary metabolites (Table 2). Among all the pathways, the cluster for ‘Limonene and pinene degradation [PATH: ko00903] represents the largest group followed by Phenylpropanoid biosynthesis [PATH: ko00940] and Isoquinoline Alkaloid Biosynthesis [PATH: ko00950] respectively. A broad survey of cellular metabolism involved in conversion of sucrose to BIAs resulted in identification of transcripts corresponding to substantial number of metabolic pathway enzymes. Analysis suggested that all the known genes involved in the biosynthesis of BIAs were present in the combined transcriptome data generated in our study (Figure S3). Number of reads which assembled to specific BIA biosynthesis related genes is provided in Figure S4. Out of all the genes, berberine bridge enzyme (BBE) had lowest number of reads (583) whereas (S)-norcoclaurine-6-O-methyltransferase (6OMT) was formed with highest number of reads (13,442). The annotated contigs homologous to genes of the primary metabolic pathways displayed high homology to Arabidopsis or other dicot genes, with most of the genes having more than 80% similarity at the protein levels, suggesting that these genes were highly conserved during the evolution.

**Table 2:** The unigenes related to secondary metabolites.

Biosynthesis of Other Secondary Metabolites	Unigene
Anthocyanin Biosynthesis [PATH:ko00942]	62
Brassinosteroid Biosynthesis [PATH:ko00905]	21
Caffeine Metabolism [PATH:ko00232]	10
Carotenoid Biosynthesis [PATH:ko00906]	38
Diterpenoid Biosynthesis [PATH:ko00904]	297
Flavonoid Biosynthesis [PATH:ko00941]	204
Flavone and Flavonol Biosynthesis [PATH:ko00944]	128
Glucosinolate Biosynthesis [PATH:ko00966]	139
Indole Alkaloid Biosynthesis [PATH:ko00901]	141
Isoquinoline Alkaloid Biosynthesis [PATH:ko00950]	360
Monoterpenoid biosynthesis [PATH:ko00902]	10
Limonene and pinene degradation [PATH:ko00903]	1185
Novabiocin Biosynthesis	174
Phenylpropanoid Biosynthesis [PATH:ko00940]	666
Sesquiterpenoid and Triterpenoid Biosynthesis [PATH:ko00909]	16
Sesquiterpenoid and Triterpenoid Biosynthesis [PATH:ko00909]	16
Terpenoid backbone Biosynthesis [PATH:ko00900]	152
Tetracycline Biosynthesis	28
Zeatin Biosynthesis [PATH:ko00908]	18
**Total**	**3665**

### Identification of differentially expressing transcript involved in papaverine biosynthesis

**Figure 6:**
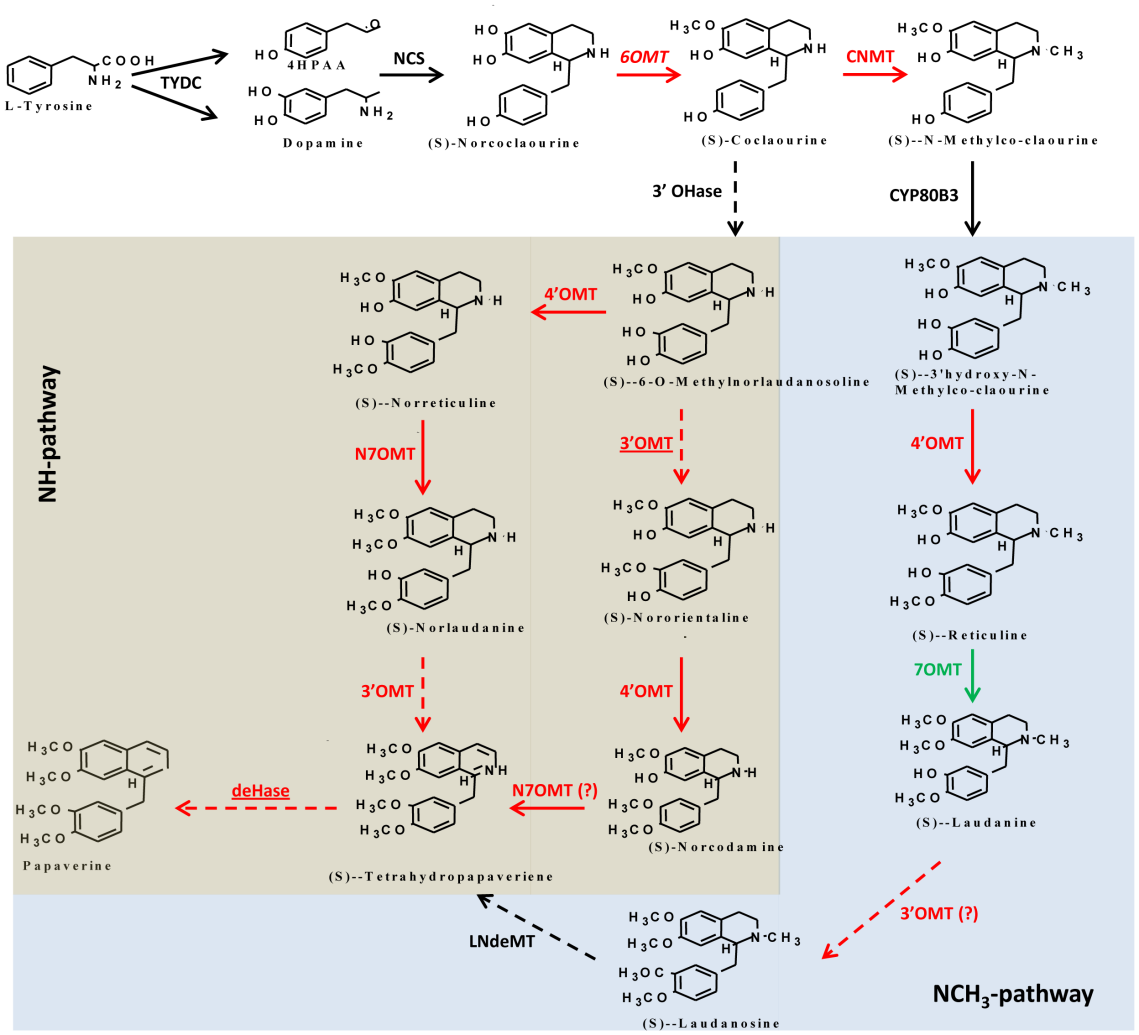
Proposed biosynthetic pathways (NH - and NCH3-pathways) and expression of genes in ***pap1*** mutant. Enzymes for which information about cDNAs is not known are shown by broken arrows. Enzymes in red show up-regulation in *pap1* in comparison to BR086, whereas in green (7OMT) shows down-regulation. Transcripts with no change in expression are shown in black. Transcripts identified through comparative transcriptome analysis with putative involvement in papaverine are underlined. Abbreviations: TYDC, tyrosine decarboxylase; NCS, norcoclaurine synthase; 6OMT, (S)-norcoclaurine 6-O-methyltransferase; CNMT, (S)-coclaurine N-methyltransferase; NMCH, (S)-N-methylcoclaurine 3′-hydroxylase; 3′OHase, 3′-hydroxylase; 4′OMT, (S)-3′-hydroxyN-methylcoclaurine 4′-O-methyltransferase; N7OMT, norreticuline 7-O-methyltransferase; 7OMT, reticuline 7-O-methyltransferase; 3′OMT, 3′-O-methyltransferase; deHase, dehydrogenase; LNdeMT, laudanosine N-demethylase.

Before our study, there have been two studies on poppy transcriptome which generated information of unigenes from poppy cell cultures (93,723 unigenes) for sanguinarine biosynthesis [27] and comparative analysis of eight different cultivars (58,140 to 111,841 unigenes) [39]. These studies were confined to identify genes involved in the pathways and no comparative analysis was carried out for elucidation of a single biochemical route of specific alkaloid. However, our comparative analysis using *pap1* and BR086 transcriptomes was aimed to identify putative genes which might be involved in papaverine biosynthesis. To identify differentially expressing genes, the R statistical test [32] was applied and only R value ≥8 was filtered that gave a believability of >99%. In this test, the singletons were statistically insignificant and hence discarded. Out of 46,920 contigs from combined assembly of *pap1* and BR086 only 3,336 were significantly (R value ≥8; fold change ≥2) differentially expressed. Of these, 1393 genes were up-regulated (Table S10) with more than 2-fold change in expression in *pap1* in comparison to BR086. Likewise 1943 genes were down-regulated (Table S11) with more than 2-fold change in expression in *pap1* in comparison to BR086. Of these, 76 up-regulated and 272 down-regulated genes did not give hits in any of the databases analyzed and were novel genes and may be involved in different pathways or molecular networks involved in primary and secondary metabolic pathways. Our analysis suggested that out of all known genes involved in BIA biosynthesis, TYDC, NCS and CYP80B3 does not express differentially between *pap1* and BR086. This suggests that substrate flux for the biosynthesis of (S)-norcoclaurine which in part of both NH- and NCH_3_-pathways (Figure 6) may not be deciding factor for the papaverine biosynthesis.

### Putative genes involved in uncharacterized steps of papaverine biosynthesis

Using different set of experiments, studies suggest two putative and controversial routes of papaverine biosynthesis (Figure 6). Both the pathway utilize (S)-norcoclaurine synthesized from precursor L-tyrosine. Out of two pathways, the NH-pathway which utilizes N-desmethylated intermediates was proposed on the basis of feeding experiments with labeled precursors combined with controlled degradation of labeled papaverine [40] and *in vitro* functional characterization of norreticuline-7-O-methyltransferase (N7OMT) [21]. The second proposed pathway (NCH_3_ pathway) proceeds through several N-methylated intermediates and involves (S)-reticuline [23]. However acceptance of both (S)-reticuline and (S)-norreticuline as substrate does not discount either the N-desmethyl or N-methyl benzylisoquinoline routes [24]. The controversy also exists due to absence of information related to genes/enzymes involved in uncharacterized steps like 3′-O-methylation and an unknown dehydrogenation required for papaverine biosynthesis. These three steps are bottlenecks in the proposed pathway and identification of genes involved in these steps will lead to elucidate exact pathway. It has been suggested that to solve this controversy, there is need to use systems biology approach utilizing mutants/contrasting chemotypes of papaverine biosynthesis, NGS technology and functional genomics. Recently, such analysis have been carried out using *top1* (thebaine oripavine poppy 1) mutant which has helped in identifying genes involved in thebaine biosynthesis [17]. In case of papaverine biosynthesis pathway several missing links need to be elucidated, these uncharacterized step involves members of methyltransferase, cytochrome P450, and dehydrogenase gene families. In our transcriptome data, we analyzed for transcripts encoding members of these gene families and studied their differential expression.

**Figure 7:**
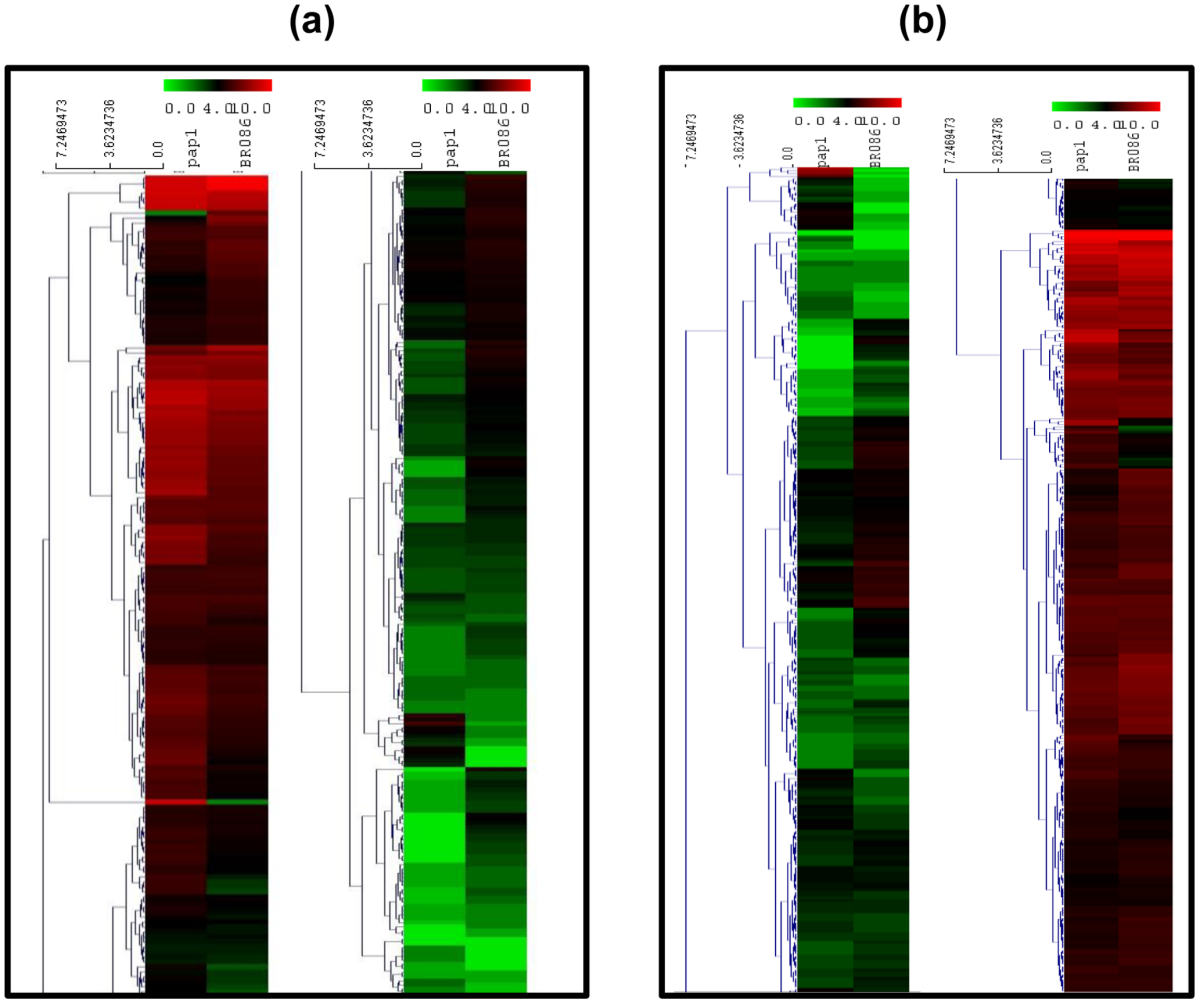
Differential expression of methyltransferases (a) and dehydrogenases (b) with their differential expression in ***pap1*** and BR086. Two columns in each represent *pap1* and BR086, while each row represents contigs encoding members of these families (Table S10 and S11). Clustering was carried out with log tpm value of each contig in *pap1* and BR086 transcriptome to visualize differential expression.

After pooling and stringent filtering of combined assembled transcriptome data, a total of 363 contigs annotated as methyltransferases were identified (Table S12). Out of these, more than 100 contigs are already reported in BIA pathways from different plants. A total of 36 contigs showed annotation with *Papaver somniferum* EST database. Several contigs were annotated as 4′OMT, 6′OMT, CNMT, 7OMT and N7OMT as well as SAM dependent methyl transferase already known in *P.somniferum*. Hierarchy clustering analysis suggest that number of MTs are differentially expressed between *pap1* and BR086 (Figure 7a). Of the total MTs, 22 and 37 are at least 2-fold up- or down-regulated in *pap1* in comparison to BR086. The up-regulated contigs also include contigs showing homology to 4′OMT (contig30047; 3.4-fold), 6′OMT (contig25475; 2.2-fold), CNMT (contig 27244; 2.3-fold) and N7OMT (contig52316; 2.1 fold) (Table S12) which are categorized in N-desmethyl group. Out of total down-regulated MTs, (contigs Nos. 26349, 42117 and 9735) were down-regulated most. In these, contig26349 encoding (R,S)-reticuline 7-O-methyltransferase (7OMT) was down-regulated (4.65-fold) which is proposed to be involved in N-CH3 pathway and utilizes (S)-reticuline (Figure 6) to synthesize papaverine [23]. As per proposed pathways, if NCH_3_-pathway is involved in enhanced biosynthesis of papaverine in *pap1,* it will require up-regulation of 7OMT to divert flux towards papaverine biosynthesis instead of BIAs. Interestingly, our analysis revealed more than 4-fold down-regulation of 7OMT in *pap1.* Collectively, up-regulation of known 6OMT, 4OMT and N7OMT as well as down-regulation of 7OMT suggests that in *pap1* mutant, (S)-reticuline may not be preferred substrate for papaverine biosynthesis. However, to utilize (S)-coclaurine via NCH_3_ pathway for papaverine biosynthesis, additional 3′OMT is required.This route of pathway needs methylation at three steps which can be catalysed by 4′OMT, N7OMT and 3′OMT. In our transcriptome data, several unigenes homologus to N7OMT showed 1.7- to 2.1-fold more expression (Table S12) in *pap1* in comparison to BR086 suggesting involvement of N7OMT in papaverine biosynthesis. Earlier, 4′OMT has been shown to be promiscuous and involvement of same enzyme was shown in NH- and NCH_3_- pathway [25]. 4′OMT with established function in biosynthesis of BIAs is up-regulated more than 3-fold in *pap1* in comparison to BR086. Together, up-regulation of N7OMT and 4′OMT suggests that that NCH_3_-pathway plays important role in papaverine biosynthesis.

We also looked for putative candidates genes encoding 3′OMT which can be part of uncharacterized step in NCH_3_-pathway. Out of 22 total up-regulated (more than 2-fold) MTs, interestingly, one unigene (contig23231) (Table S12) showed maximum homology to 9-O-methyltransferase (9OMT) from *Thalictrum tuberosum* is up-regulated more than 5-fold (Table S12; Figure 7a). Recently, SOMT1 annotated as 9-O-methyltransferase accept both (S)-norreticuline or (S)-reticuline as substrate. This SOMT1 is the only O-methyltransferase which functions efficiently on both N-methylated and N-desmethylated benzyl isoquinoline. SOMT1 gene shows 3′-O-methyltransferase activity and silencing of SOMT1 reduce papaverine level [24]. As contig23231 shows homology to SOMT1, we conclude that transcript related to this contig might be encoding for 3′OMT and participate in papaverine biosynthesis via NCH_3_ route.

In our transcriptome data, a total of 584 dehydrogenases were present (Table S13) which falls into short chain Dehydrogenase/reductase (SDR) families including tropionine reductase, alcohol dehydrogenase and malate dehydrogenase. Hierarchy clustering analysis suggest that number of dehydrogenases are differentially expressed between *pap1* and BR086 (Figure 7b). N-desmethyl route of papaverine biosynthesis involved uncharacterized dehydrogenase which converts (S)-tertahydropapaverine to papaverine (Figure 6). Of the total dehydrogenases, 34 and 33 are at least 2-fold up- or down-regulated in *pap1* in comparison to BR086. The up-regulated dehydrogenases identified in this study, might be involved in the uncharacterized step leading to papaverine biosynthesis from (S)-tetrahydropapaverine (Figure 6).

### Validation of expression of differentially expressed genes

**Figure 8:**
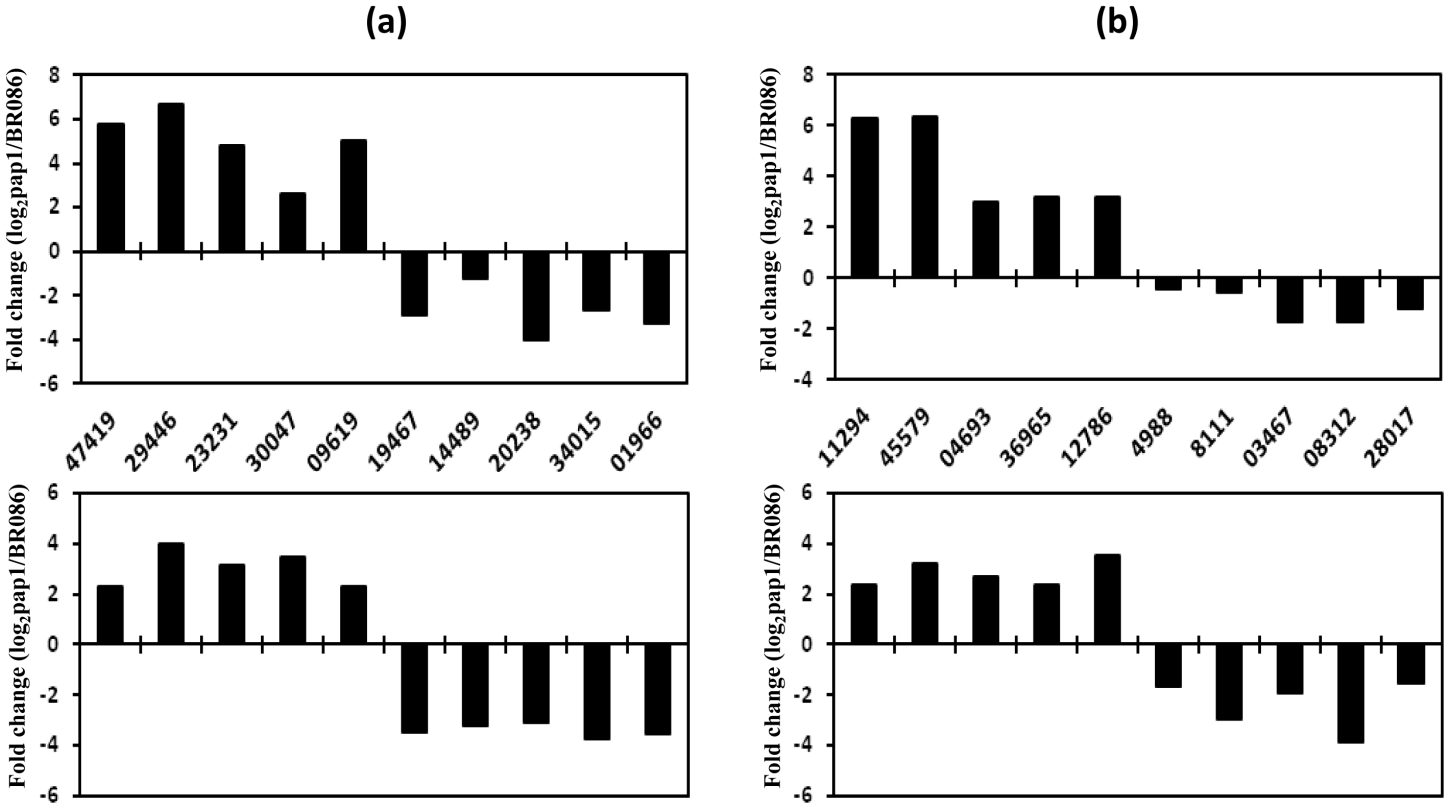
Validation of differentially expressed transcripts in ***pap1*** and BR086. Real time quantitative PCR of selected differentially regulated methyltransferase (a) and dehydrogenase gene family (b) was carried out using total RNA isolated from peduncle tissue of prehook stage of *pap1* mutant and BR086. Digital gene expression of genes used for validation through quantitative Real time PCR is provided in lower panel of (a) and (b). Information about selected up-and down-regulated transcripts is provided in Table S1.

Analysis of transcriptome data from *pap1* and BR086, identified genes encoding members of methyl transferase and dehydrogenase gene families which might be involved in papaverine biosynthesis. To validate differential expression of these identified genes, we selected 10 methyl transferases (5 up- and 5-down-regulated) and 10 dehydrogenases family (5 up- and 5-down-regulated) members for validation of expression. All these genes showed differential expression (up and down-regulation) in *pap1* in comparison to BR086 (Figure 8a and 8b; upper panel). The differential expression observed was similar to digital expression results (Figure 8a and 8b; lower panel) suggesting that identified genes, apart from those taken for validation through real-time PCR, are differentially expressed between *pap1* and BR086. Similar analysis through analysis of transcriptomes from different tissues for differentially expressed genes has led to the identification of genes involved in secondary plant product biosynthesis [41]. The validation of expression of selected genes through real-time PCR suggests that other differentially expressed genes identified, in this study, through digital expression analysis may be involved in various uncharacterized steps in papaverine biosynthesis.

## Conclusion

Biosynthetic pathways for paparverine production in *Papaver somnifera* have only been putative and controversial; our study attempted to rectify this discrepancy through transcriptional profiling in a high-papaverine producing mutant, *pap1*, compared to wild-type, BR086. Our comparative transcriptome analysis clearly suggests that papaverine biosynthesis does not primarily proceed through the N-demethylated branch-point intermediate (S)-reticuline as proposed one of the pathways. Instead, biosynthesis of papaverine appears to utilize (S)-coclaurine as a key branch-point intermediate. Our analysis also identifies a set of methyl transferases and dehydrogenases which might be playing role in uncharacterized steps in papaverine biosynthesis through NCH_3_-pathway. Functional characterization of identified genes in this study will lead to establish papaverine biosynthetic pathway which has been controversial for a long time.

## Supporting Information

Figure S1 **Gene ontology classification of** ***pap1*** **and BR086 transcripts.** BLASTX in Arabidopsis (TAIR) proteome was carried out to provide the GO annotation. The results are summarized in three main categories: Biological Process (BP), Cellular Component (CC) and Molecular Function (MF).
(TIF)

Figure S2 **Gene ontology classification of unigenes from combined assembly.** BLASTX in Arabidopsis (TAIR) proteome was carried out to provide the GO annotation. The results are summarized in three main categories: Biological Process, Cellular Component and Molecular Function.
(TIF)

Figure S3 **Biosynthetic pathway of different BIAs and representation of transcripts encoding known genes in combined assembled transcriptome.** Enzymes shown in red are represented in transcriptome data generated in present study.
(TIF)

Figure S4 **Number of high-quality reads representing known genes of BIA pathway in combined assembly.** Known genes for which analysis was carried out include TYDC, tyrosine/dopa decarboxylase; NCS, norcoclaurine synthase; CNMT, (S)-coclaurine N-methyltransferase; 4′OMT, (S)-3′-hdroxy-N-methylcoclaurine 4′-O-methyltransferase; 6OMT, (S)-norcoclaurine-6-O-methyltransferase; N7OMT, norreticuline 7-*O*methyltransferase; 7OMT, reticuline 7-*O*-methyltransferase; SalR salutaridine reductase; SalAT, salutaridinol 7-O-acetyltransferase ; T6ODM, thebaine 6-O-demethylase; CODM, codeine O-demethylase; BBE, berberine bridge enzyme and COR, Codionine reductase.
(TIF)

Table S1 **List of different contigs, encoded gene families and primers used for validation of expression.**
(DOC)

Table S2 **Annotation of unigenes of** ***pap1*** **using NR database.**
(XLS)

Table S3 **Annotation of unigenes of** ***pap1*** **using TAIR database.**
(XLS)

Table S4 **Annotation of unigenes of pap1 using CDD database.**
(XLS)

Table S5 **Annotation of unigenes of BR086 using NR database.**
(XLS)

Table S6 **Annotation of unigenes of BR086 using CDD database.**
(XLS)

Table S7 **Annotation of unigenes of BR086 using TAIR database.**
(XLS)

Table S8 **Summary of annotation of unigenes using different databases.**
(DOC)

Table S9 **Details of unigenes involved in other metabolic processes.**
(XLS)

Table S10 **List of up-regulated unigenes in** ***pap1*** **in comparison to BR086.**
(XLS)

Table S11 **List of down-regulated unigenes in** ***pap1*** **in comparison to BR086.**
(XLS)

Table S12 **List of methyltranferases identified in** ***pap1*** **and BR086 and their relative expression.**
(XLS)

Table S13 **List of dehydrogenases identified in** ***pap1*** **and BR086 and their relative expression**
(XLS)

## References

[1] Sato Y, He JX, Nagai H, Tani T, Akao T (2007) Isoliquiritigenin, one of the antispasmodic principles of *Glycyrrhiza ularensis* roots, acts in the lower part of intestine. Biol Pharma Bull 30: 145–149. 10.1248/bpb.30.145 17202675

[2] Thomas JA (2002) Pharmacological aspects of erectile dysfunction. Jap J Pharma 89: 101–112. 10.1254/jjp.89.101 12120751

[3] Brisman JL, Eskridge JM, Newell DW (2006) Neuro interventional treatment of vasospasm. Neurol Res 28: 769–776. 17164040 10.1179/016164106X152043

[4] Damen L, Bruijn J, Verhagen AP, Berger MY, Passchier J, et al (2006) Prophylactic treatment of migraine in children. Part 2. Asystematic review of pharmacological trials. Cephalagia 26: 497–505. 10.1111/j.1468-2982.2005.01047.x 16674757

[5] Menniti FS, Chappie TA, Humphrey JM, Schmidt CJ (2007) Phosphoesterase 10 A inhibiters: A novel approach to the symptoms of schizophrenia. Curr Opin Invest Drugs 8: 54–59. 17263185

[6] Boswell-Smith V, Spina D, Page CP (2006) Phosphodiesterase inhibitors Bri J Pharmacol 147: : S252–S257. 10.1038/sj.bjp.0706495 PMC1760738 16402111

[7] Liscombe DK, MacLeod BP, Loukanina N, Nandi OI, Facchini PJ (2005) Evidence for the monophyletic evolution of benzylisoquinoline alkaloid biosynthesis in angiosperms. Phytochemisty 66: 2501–2520. 10.1016/j.phytochem.2005.04.044 16342378

[8] Lee EJ, Facchini PJ (2010) Norcoclaurine synthase is a member of the pathogenesis-related 10/Bet v1 protein family. Plant Cell 22: 3489–3503. 21037103 10.1105/tpc.110.077958 PMC2990142

[9] Pauli HH, Kutchan TM (1998) Molecular cloning and functional heterologous expression of two alleles encoding (S)-N-methylcoclaurine 3′-hydroxylase (Cyp80b1), a new methyl jasmonate-inducible cytochrome P-450-dependent mono-oxygenase of benzylisoquinoline alkaloid biosynthesis. Plant J 13: 793–801. 9681018 10.1046/j.1365-313x.1998.00085.x

[10] Facchini PJ, Park SU (2003) Developmental and inducible accumulation of gene transcripts involved in alkaloid biosynthesis in opium poppy. Phytochemistry 64: 177–186. 12946416 10.1016/s0031-9422(03)00292-9

[11] Ziegler J, Diaz-Chávez M, Kramell R, Ammer C, Kutchan TM (2005) Comparative macroarray analysis of morphine containing *Papaver somniferum* and eight morphine free Papaver species identifies an O-methyltransferase involved in benzylisoquinoline biosynthesis. Planta 222: 458–471. 16034588 10.1007/s00425-005-1550-4

[12] Gerardy R, Zenk MH (1993) Formation of salutaridine from (R)- reticuline by a membrane bound cytochrome P450 enzyme from *Papaver somniferum* . Phytochemistry 32: 79–86.

[13] Unterlinner B, Lenz R, Kutchan TM (1999) Molecular cloning and functional expression of codeinone reductase: the penultimate enzyme in morphine biosynthesis in the opium poppy *Papaver somniferum* . Plant J 18: 465–475. 10417697 10.1046/j.1365-313x.1999.00470.x

[14] Hirata K, Poeaknapo C, Schmidt J, Zenk MH (2004) 1, 2-Dehydroreticuline synthase, the branch point enzyme opening the morphinan biosynthetic pathway. Phytochemistry 65: 1039–1046. 15110683 10.1016/j.phytochem.2004.02.015

[15] Ziegler J, Voigtländer S, Schmidt J, Kramell R, Miersch O, et al (2006) Comparative transcript and alkaloid profiling in Papaver species identifies a short chain dehydrogenase/reductase involved in morphine biosynthesis. Plant J 48: 177–192. 16968522 10.1111/j.1365-313X.2006.02860.x

[16] Gesell A, Rolf M, Ziegler J, Díaz-Chávez ML, Huang FC, et al (2009) CYP719B1 is salutaridine synthase, the C–C phenolcoupling enzyme of morphine biosynthesis in opium poppy. J Biol Chem 284: 24432–24442. 19567876 10.1074/jbc.M109.033373 PMC2782036

[17] Hagel JM, Facchini PJ (2010) Dioxygenases catalyze the O-demethylation steps of morphine biosynthesis in opium poppy. Nat Chem Biol 6: 273–275. 20228795 10.1038/nchembio.317

[18] Penna DD, O’Connor SE (2012) Plant Gene Clusters and Opiates. Science 336: 1648. 22745410 10.1126/science.1225473

[19] Brochmann-Hanssen E, Leung AY, Fu CC, Zanati G (1971) Opium alkaloids X. Biosynthesis of benzylisoquinolines. J Pharmocol Sci 60: 1672–1676. 10.1002/jps.2600601118 5133918

[20] Brochmann-Hanssen E, Chen CH, Chen CR, Chiang HC, Leung AY, et al (1975) Opium alkaloids. Part XVI. The biosynthesis of benzylisoquinolines in *Papaver somniferum*. Preferred and secondary pathways; stereo chemical aspects. J Chem Soc 16: 1531–1537. 10.1039/p19750001531 1236862

[21] Pienkny S, Brandt W, Schmidt J, Kramell R, Ziegler J (2009) Functional characterization of a novel benzylisoquinoline-O-methyltransferase suggests its involvement in papaverine biosynthesis in opium poppy (*Papaver somniferum* L). Plant J 60: 56–67. 19500305 10.1111/j.1365-313X.2009.03937.x

[22] Ounaroon A, Decker G, Schmidt J, Lottspeich F, Kutchan TM (2003) (R, S)-Reticuline 7-O-methyltransferase and (R, S)-norcoclaurine 6-O-methyltransferase of *Papaver somniferum*- cDNA cloningand characterization of methyl transfer enzymes of alkaloid biosynthesis in opium poppy. Plant J 36: 808–819. 14675446 10.1046/j.1365-313x.2003.01928.x

[23] Han X, Lamshoft M, Grobe N, Ren X, Fist AJ, et al (2010) The biosynthesis of papaverine proceeds via (S)-reticuline. Phytochemistry 71: 1305–1312. 20494383 10.1016/j.phytochem.2010.04.022 PMC2900451

[24] Dang TT, Facchini PJ (2012) Characterization of three O-methyltransferases involved in noscapine biosynthesis in opium poppy. Plant Physiol 159: 618–631. 22535422 10.1104/pp.112.194886 PMC3375929

[25] Desgagné-Penix I, Facchini PJ (2012) Systematic silencing of benzylisoquinoline alkaloid biosynthetic genes reveals the major route to papaverine in opium poppy. Plant J 72: 331–344. 22725256 10.1111/j.1365-313X.2012.05084.x

[26] Ziegler J, Facchini PJ (2008) Alkaloid biosynthesis: metabolism and trafficking. Annu Rev Plant Biol 59: 735–769. 18251710 10.1146/annurev.arplant.59.032607.092730

[27] Desgagné-Penix I, Khan MF, Schriemer DC, Nowak J, Facchini PJ (2010) Integration of deep transcriptome and proteome analyses reveals the components of alkaloid metabolism in opium poppy cell cultures. BMC Plant Biol 10: 252–268. 21083930 10.1186/1471-2229-10-252 PMC3095332

[28] Luca VD, Salim V, Atsumi SM, Yu F (2012) Mining the biodiversity of plants: a revolution in the making. Science 336: 1658. 22745417 10.1126/science.1217410

[29] Millgate AG, Pogson BJ, Wilson IW, Kutchan TM, Zenk MH, et al (2004) Analgesia: morphine-pathway block in top1 poppies. Nature 431: 413–414. 15386001 10.1038/431413a

[30] Ashburner M, Ball CA, Blake JA, Botstein D, Butler H, et al (2000) Gene ontology: tool for the unification of biology. Nat Genet 25: 25–29. 10802651 10.1038/75556 PMC3037419

[31] Moriya Y, Itoh M, Okuda S, Yoshizawa AC, Kanehisa M (2007) KAAS: an automatic genome annotation and pathway reconstruction server. Nucl Acids Res 35: W182–W185. 17526522 10.1093/nar/gkm321 PMC1933193

[32] Stekel D J, Git Y, Falciani F (2000) The Comparison of Gene Expression from Multiple cDNA Libraries. Genome Res 10: 2055–2061. 11116099 10.1101/gr.gr-1325rr PMC313085

[33] Eisen MB, Spellman PT, Brown PO, Botstein D (1998) Cluster analysis and display of genome wide expression patterns. Proc Natl Acad Sci USA 95: 14863–14868. 9843981 10.1073/pnas.95.25.14863 PMC24541

[34] Mishra BK, Pathak S, Sharma A, Trivedi PK, Shukla S (2010) Modulated gene expression in newly synthesized auto-tetraploid of *Papaver somniferum L.* . S Afr J Bot 76: 447–452.

[35] Pathak S, Mishra BK, Misra P, Misra P, Joshi VK, et al (2012) High frequency somatic embryogenesis, regeneration and correlation of alkaloid biosynthesis with gene expression in *Papaver somniferum* . Plant Growth Regul 68: 17–25.

[36] Khanna KR, Shukla S (1991) Inheritance of papaverine in *Papaver somniferum* L. and a morphological marker for high papaverine plants. Herba Hungarica 30: 7–10.

[37] Shukla S, Singh SP (1999) Genetic systems involved in the inheritance of papaverine in opium poppy (Papaver somniferum L.). Ind J Agri Sci 69: 44–47.

[38] Weid M, Ziegler J, Kutchan TM (2004) The roles of latex and the vascular bundle in morphine biosynthesis in the opium poppy, *Papaver somniferum* . Proc Natl Acad Sci USA 101: 13957–13962. 15353584 10.1073/pnas.0405704101 PMC518766

[39] Desgagné-Penix I, Farrow SC, Cram D, Nowak J, Facchini PJ (2012) Integration of deep transcript and targeted metabolite profiles for eight cultivars of opium poppy. Plant Mol Biol 3: 295–313. 10.1007/s11103-012-9913-2 22527754

[40] Brochmann-Hanssen E, Chen CY, Linn EE (1980) Biosynthesis of unnatural papaverine derivatives in *Papaver somniferum* . J Nat Prod 43: 736–738. 20707397 10.1021/np50012a007

[41] Gupta P, Goel R, Pathak S, Srivastava A, Singh SP, et al. (2013) De novo assembly, functional annotation and comparative analysis of *Withania somnifera* leaf and root transcriptomes to identify putative genes involved in the withanolides biosynthesis. PLOS ONE (In Press) DOI: 10.1371/journal.pone.0062714. 10.1371/journal.pone.0062714 PMC3648579 23667511

