# Supplementary figures and images for: Comparative Transcriptome Analysis Using High Papaverine Mutant of Papaver somniferum Reveals Pathway and Uncharacterized Steps of Papaverine Biosynthesis
Source: PLoS One. 2013 May 30;8(5):e65622. doi: 10.1371/journal.pone.0065622 (PMC3667846; doi:10.1371/journal.pone.0065622)

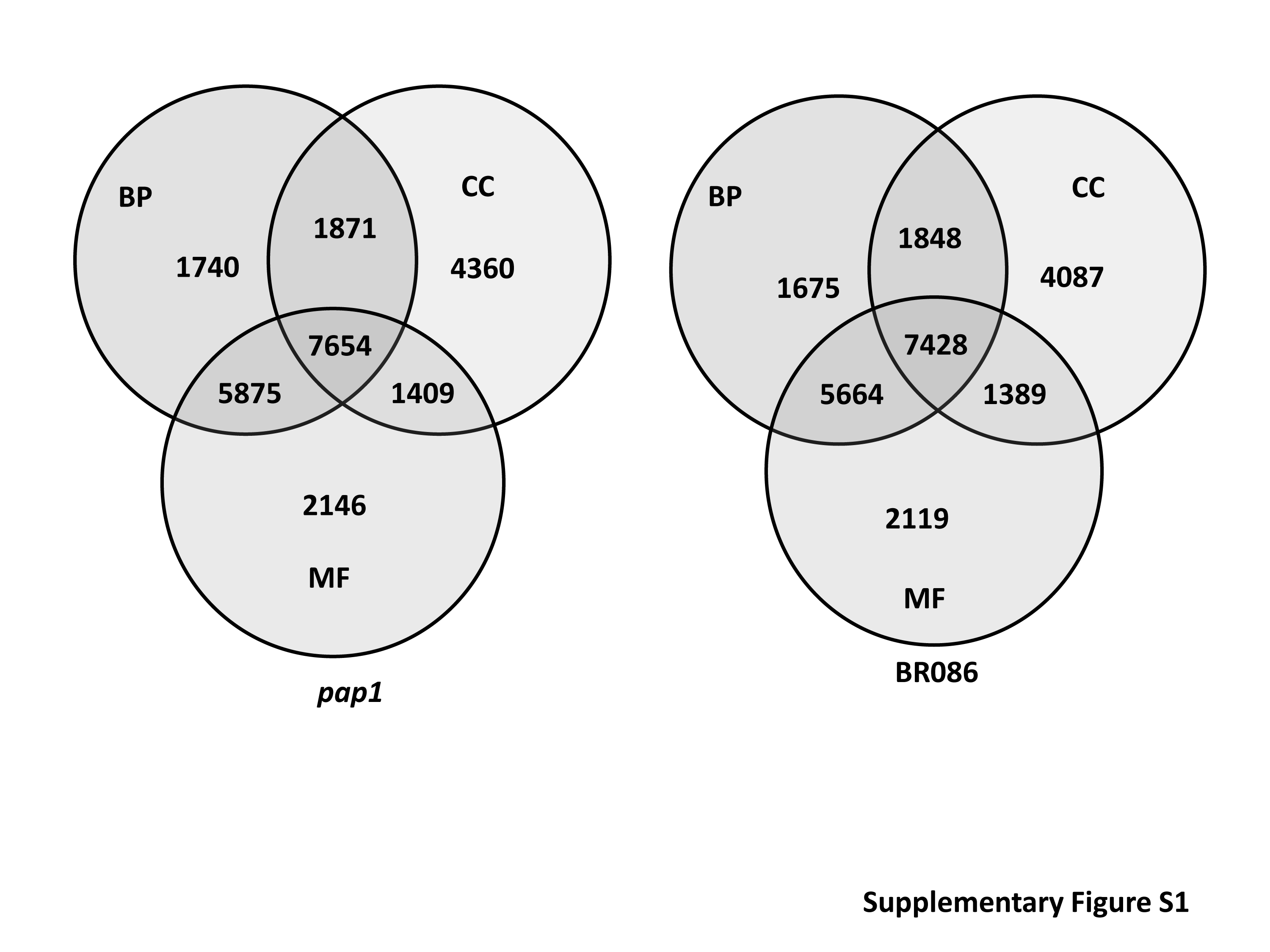

Supplement: Figure S1 — Gene ontology classification of pap1 and BR086 transcripts. BLASTX in Arabidopsis (TAIR) proteome was carried out to provide the GO annotation. The results are summarized in three main categories: Biological Process (BP), Cellular Component (CC) and Molecular Function (MF). (TIF) [file pone.0065622.s001.tif]

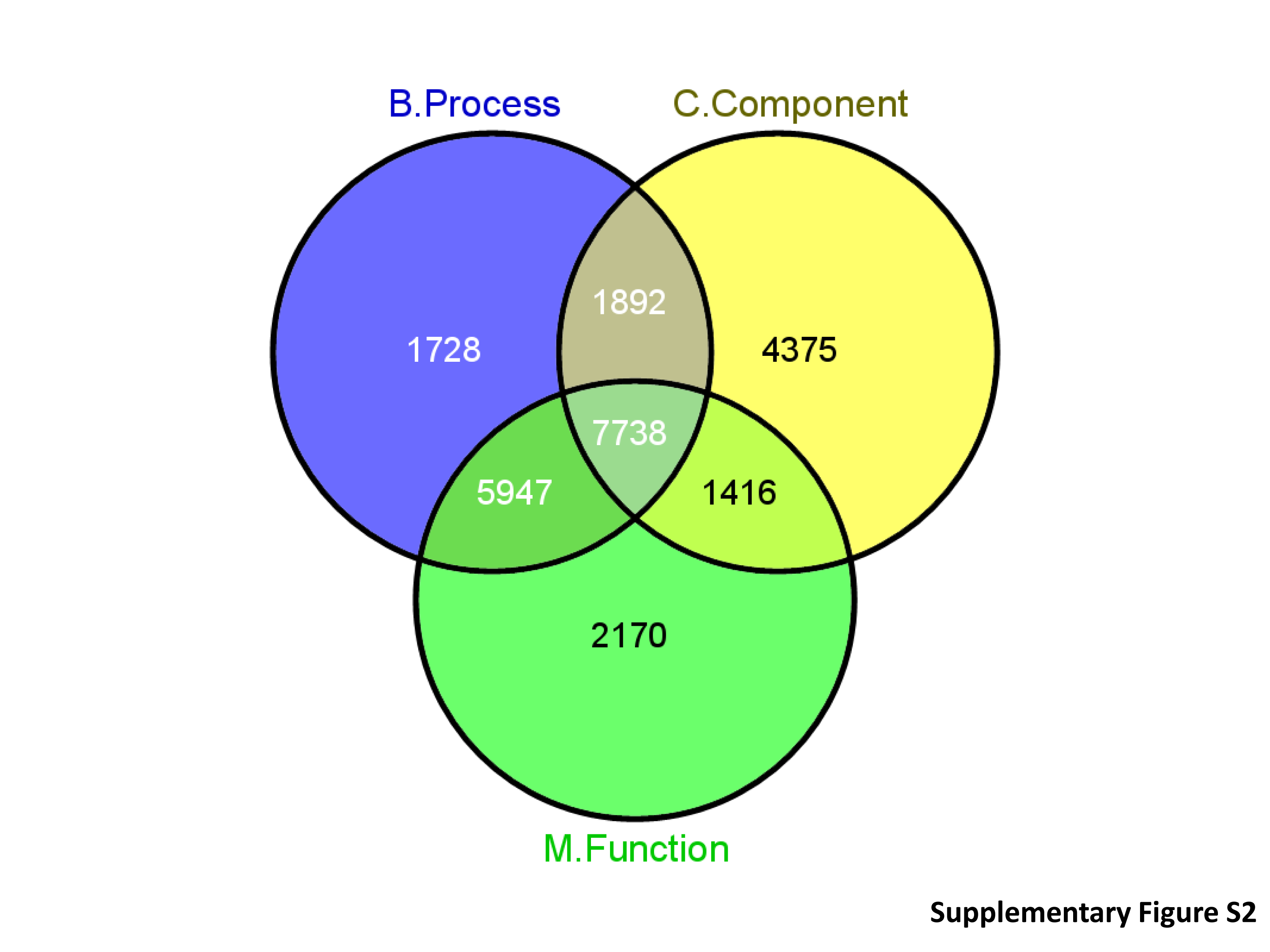

Supplement: Figure S2 — Gene ontology classification of unigenes from combined assembly. BLASTX in Arabidopsis (TAIR) proteome was carried out to provide the GO annotation. The results are summarized in three main categories: Biological Process, Cellular Component and Molecular Function. (TIF) [file pone.0065622.s002.tif]

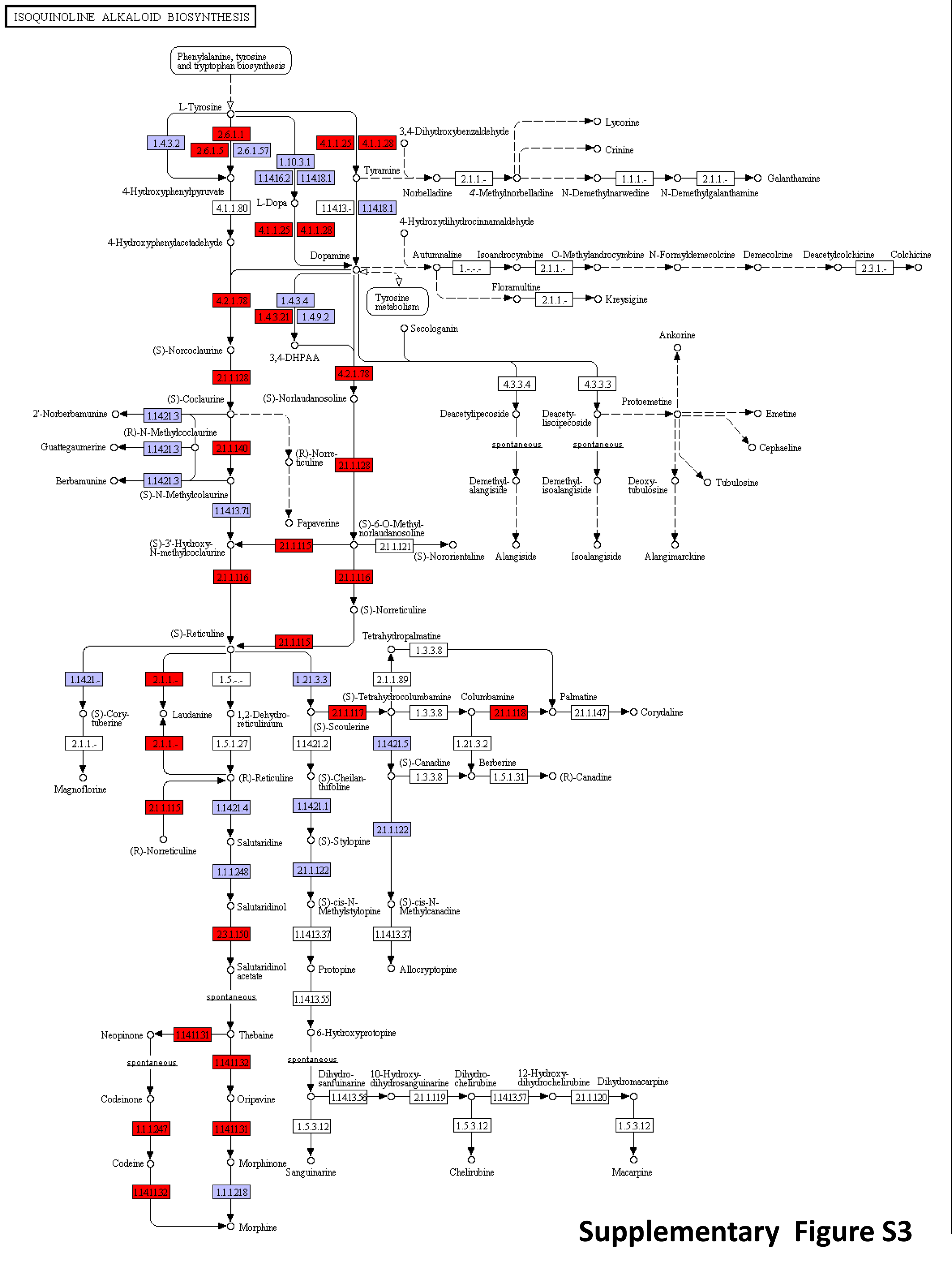

Supplement: Figure S3 — Biosynthetic pathway of different BIAs and representation of transcripts encoding known genes in combined assembled transcriptome. Enzymes shown in red are represented in transcriptome data generated in present study. (TIF) [file pone.0065622.s003.tif]

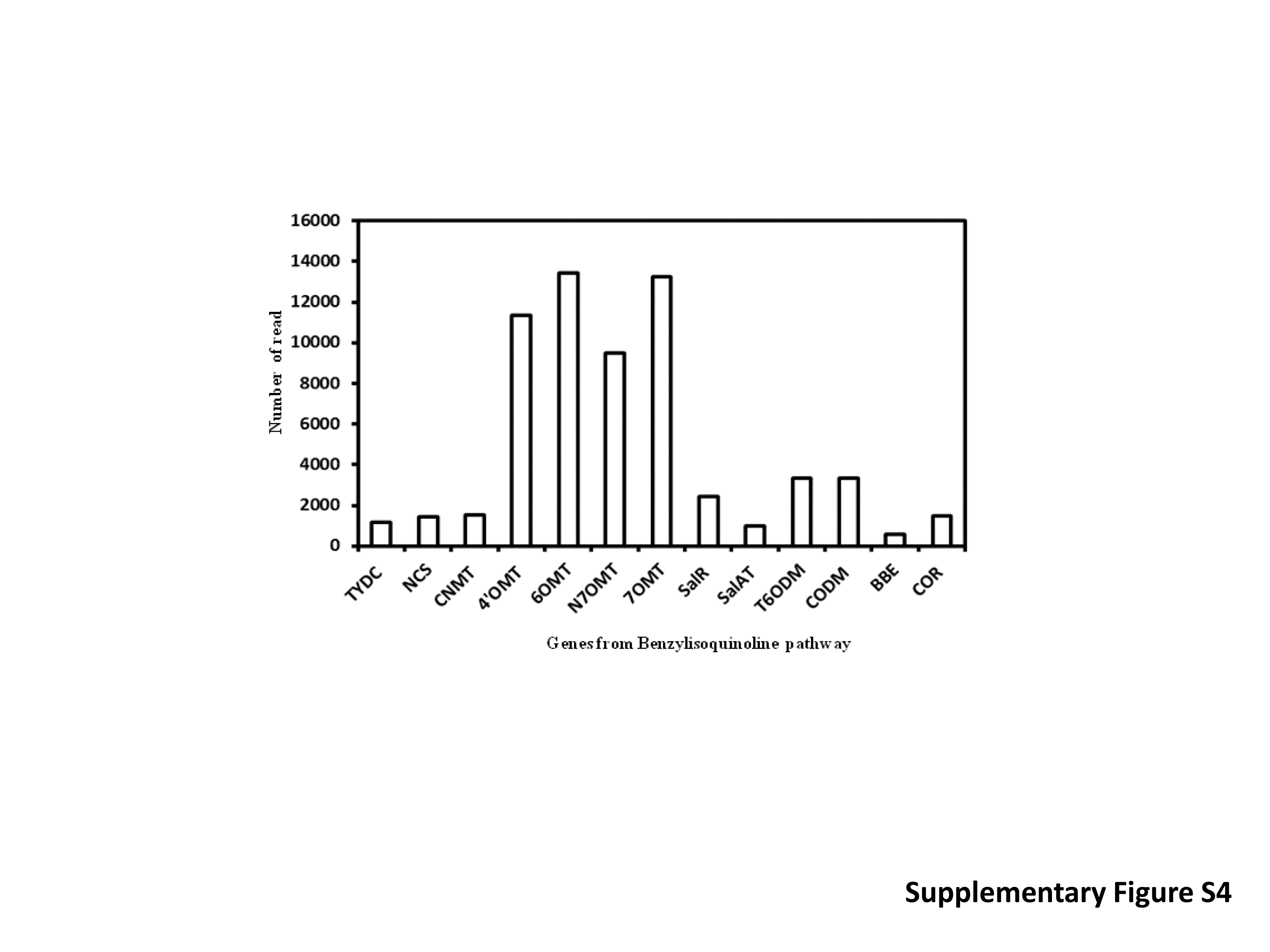

Supplement: Figure S4 — Number of high-quality reads representing known genes of BIA pathway in combined assembly. Known genes for which analysis was carried out include TYDC, tyrosine/dopa decarboxylase; NCS, norcoclaurine synthase; CNMT, (S)-coclaurine N-methyltransferase; 4′OMT, (S)-3′-hdroxy-N-methylcoclaurine 4′-O-methyltransferase; 6OMT, (S)-norcoclaurine-6-O-methyltransferase; N7OMT, norreticuline 7-Omethyltransferase; 7OMT, reticuline 7-O-methyltransferase; SalR salutaridine reductase; SalAT, salutaridinol 7-O-acetyltransferase ; T6ODM, thebaine 6-O-demethylase; CODM, codeine O-demethylase; BBE, berberine bridge enzyme and COR, Codionine reductase. (TIF) [file pone.0065622.s004.tif]
