# Supplementary material for: Comparative Transcriptome Analysis Using High Papaverine Mutant of Papaver somniferum Reveals Pathway and Uncharacterized Steps of Papaverine Biosynthesis
Source: PLoS One. 2013 May 30;8(5):e65622. doi: 10.1371/journal.pone.0065622 (PMC3667846; doi:10.1371/journal.pone.0065622)
Supplement: Table S1 — List of different contigs, encoded gene families and primers used for validation of expression. (DOC) [file pone.0065622.s005.doc]

| **Contig** | **Forward** | **Reverse** | |
| --- | --- | --- | --- |
| **Methyltransferase** | | | |
| 20238 | CGGACCGTGTTTCGCTGATGGA | ACAGGCCAGAGGTACTGCAACGA | |
| 01966 | GCTGTTGAAACCAGATGGGGAGCTC | CCCCAGGGATGCAAAACCTCGT | |
| 23231 | TGGCTAGTGCATCAGTACCAGTGA | ACCTGCGATGCAATATACGCAGCA | |
| 30047 | AAGCTCGGGGAAACAAACGTCCA | GGGACAGACCTTAAGCAAACTGCGA | |
| 14489 | ATGTGGCCCTCTTTGCAAGTGTCC | AAGGATCTGACTCCCCATCCCCTT | |
| 19467 | TCTCAGGGAGAAGTTACGGAGGAGA | TTGGATCGCCCTGACAGCCA | |
| 29446 | TGGTAGCTTTGGAGCGCATCTC | AGCGAAAGACCGATCATTGCAGG | |
| 34015 | TGAAGAGGTTTTGCATCCCTGGGGA | AGACACCTCTTCCATCTCCCAAGCT | |
| 09619 | TCTCCACTGGTTATCTCAGGCTCCT | | TGCCTACAATACGCTTCAACCACAG |
| 47419 | TCGAAAGTTGATAACGGATTCGGAT | | TGCTCCTGAAGCCTCCTGTAACCC |
| **Dehydrogenase** | | | |
| 45579 | TCAAGAGAGGGTGCGCAAGTGT | | GGACTTCACCGCGGAGGATCACA |
| 36965 | GCCAACGGTACTGGAGCAATCG | | GCTTTCGCTGCTGCCACTCTCC |
| 12786 | TGCAGGGTCGTTTGTCTGTTTGGC | | ACAGCAATGGAATGCCAATCCTCAG |
| 11294 | AAGAGCCAAGTCATGAGGTGGTGT | | GCAAAAGCTGCTGGACTTTCGGA |
| 04988 | ACAGGCCAAGTGAGGTGTCCCAT | | CGTCCCCTGAGTTCCCCGTT |
| 4693 | AGCACGGCTAATGGTTGCACGA | | TCTTGCGAGAGCGCAAAGAGGA |
| 3467 | ATGGTTGTTGTGACCGTGGTGGG | | AAGGCTTTGGCTTGAGCAGCAGA |
| 08111 | TGTTGGACCAGCTGATCACTTGAG | | TCCGCCACTAGCTGCTTCAATGT |
| 28017 | GTTGCTCCTCTCTCCCTTGGGCTC | | CCTTGCCCTCTCGCCTTCAAAC |
| 08312 | ACCCTTCGCACCATCGTTCTTGT | | ACCCTTCGCACCATCGTTCTTGT |

**Supplementary Table S1: List of different contigs, encoded gene families and primers used for validation of expression**
