# Supplementary material for: Comparative Transcriptome Analysis Using High Papaverine Mutant of Papaver somniferum Reveals Pathway and Uncharacterized Steps of Papaverine Biosynthesis
Source: PLoS One. 2013 May 30;8(5):e65622. doi: 10.1371/journal.pone.0065622 (PMC3667846; doi:10.1371/journal.pone.0065622)
Supplement: Table S8 — Summary of annotation of unigenes using different databases. (DOC) [file pone.0065622.s012.doc]

| **Unigenes** | **NR** | **TAIR** | **CDD** |
| --- | --- | --- | --- |
| **Contigs** | **22821** | **22213** | **17600** |
| **Singletons** | **36617** | **35280** | **16181** |
| **Total** | **59438** | **57493** | **33781** |

**Supplementary Table S8: Summary of annotation of unigenes using different databases**
